# Reduced blood flow by laser speckle flowgraphy after ^125^I-plaque brachytherapy for uveal melanoma

**DOI:** 10.1186/s12886-022-02505-9

**Published:** 2022-06-28

**Authors:** Michelle R. Tamplin, Jui-Kai Wang, Anthony H. Vitale, Ryuya Hashimoto, Mona K. Garvin, Elaine M. Binkley, Daniel E. Hyer, John M. Buatti, H. Culver Boldt, Randy H. Kardon, Isabella M. Grumbach

**Affiliations:** 1 Department of Radiation Oncology, University of Iowa, Iowa City, IA USA; 2 Iowa City VA Center for the Prevention and Treatment of Visual Loss, Iowa City, IA USA; 3 Department of Electrical and Computer Engineering, University of Iowa, Iowa City, IA USA; 4 Department of Ophthalmology and Visual Sciences, Division of Neuro-Ophthalmology, University of Iowa, Iowa City, IA 52242 USA; 5 Department of Internal Medicine, Division of Cardiovascular Medicine, Abboud Cardiovascular Research Center, Carver College of Medicine, University of Iowa, Iowa City, IA 52242 USA

**Keywords:** Retinal blood flow, Laser speckle flowgraphy, Radiation retinopathy, Uveal melanoma

## Abstract

**Background:**

To determine whether reductions in retinal and choroidal blood flow measured by laser speckle flowgraphy are detected after ^125^I-plaque brachytherapy for uveal melanoma.

**Methods:**

In a cross-sectional study, retinal and choroidal blood flow were measured using laser speckle flowgraphy in 25 patients after treatment with ^125^I-plaque brachytherapy for uveal melanoma. Flow was analyzed in the peripapillary region by mean blur rate as well as in the entire image area with a novel superpixel-based method. Relationships between measures were determined by Spearman correlation.

**Results:**

Significant decreases in laser speckle blood flow were observed in both the retinal and choroidal vascular beds of irradiated, but not fellow, eyes. Overall, 24 of 25 patients had decreased blood flow compared to their fellow eye, including 5 of the 6 patients imaged within the first 6 months following brachytherapy. A significant negative correlation between blood flow and time from therapy was present.

**Conclusions:**

Decreases in retinal and choroidal blood flow by laser speckle flowgraphy were detected within the first 6 months following brachytherapy. Reduced retinal and choroidal blood flow may be an early indicator of microangiographic response to radiation therapy.

## Background

For patients with medium-sized melanomas, globe-sparing treatment with ^125^I-plaque brachytherapy achieves local tumor control in 95% or more cases [1, 2]. However, a progressive loss of vascularity in the superficial and deep retinal capillary plexuses, as well as enlargement of the foveal avascular zone, has been observed following ^125^I-plaque brachytherapy in the majority of patients within 3–5 years of their therapy [3, 4]. Microvasculopathy of the retina, choroid, and optic nerve head have been associated with significant vision loss [5–7]. A significant loss of capillary density, microhemorrhages, and other microvascular changes have been reported by optical coherence tomography-angiography (OCT-A) in a majority of patients 2 years or more after receiving more than 80 Gy to the fovea [8]. OCT-A imaging at earlier time points primarily shows enlargement of the foveal avascular zone with normal surrounding capillary density [3, 4, 9, 10]. We recently reported reduced capillary density by OCT-A imaging in just 4 of 13 patients seen within 24 months of brachytherapy [11], even with novel deep learning-based image analyses designed to identify subtle changes in vascularity [12]. One of the technical limitations of current clinical OCT-A is that it only differentiates between areas of no-flow of red blood cells from areas with any degree of flow. It currently cannot accurately detect decreases in blood flow velocity [13] other than showing a decrease in apparent capillary density due to no flow. Given that multiple animal studies of radiation microvasculopathy have described an early decrease in blood flow within hours to days of radiation exposure in skin and parotid glands [14–16], we hypothesized that quantitative imaging of retinal and choroidal flow could be leveraged to detect microvasculopathy at early time points in humans, when mitigation efforts may be more effective and potentially more clinically impactful.

We used laser speckle flowgraphy (LSFG), a noninvasive measure of dynamic blood flow, to quantify early reductions in retinal and choroidal blood flow following ^125^I-plaque brachytherapy for uveal melanoma. LSFG is based on the ability of moving red blood cells to blur and reduce the contrast of an infrared laser speckle pattern imaged onto the plane of the retina. Its output parameter, mean blur rate (MBR), is linearly proportional to relative blood flow velocity at each pixel imaged by a video camera sensor. LSFG is an established method of detecting blood flow variations in ocular disorders such as glaucoma [17] and ischemic optic neuropathy [18], as well as systemic conditions such as hypertension [19] and metabolic syndrome [20]. Here, we deployed LSFG to test whether reductions in blood flow velocity could be detected in patients at various time points after treatment with ^125^I-plaque brachytherapy.

## Methods

### Study participants

Fifty eyes of 25 patients with uveal melanoma treated with ^125^I-plaque brachytherapy were studied. Patients were consecutively enrolled from the Retina and Vitreous Clinic at the University of Iowa Hospital & Clinics as part of a previously reported study [11]. All participants provided written informed consent before screening or initiation of any procedures. The study protocol was approved by the University of Iowa Institutional Review Board for Human Use and followed the tenets of the Declaration of Helsinki. In this cross-sectional study, enrolled participants were imaged by LSFG at the time of their return clinic visit, which ranged from 2 weeks to 12 years following treatment with ^125^I-plaque brachytherapy. All imaging was performed after participants rested in a dark room for 10 min, until mydriasis was achieved.

### Acquisition and analysis of LSFG scans

Prior to scan acquisition, intraocular pressure (IOP) was measured by applanation tonometry (Tono-Pen XL Tonometer, Reichert Inc.) after administration of a topical anesthetic (proparacaine hydrochloride ophthalmic solution 0.5%, Alcon). Mydriasis was induced with 1% tropicamide (Akorn, Inc.) and 2.5% phenylephrine (Paragon BioTeck, Inc.). Systemic blood pressure was measured with an automatic sphygmomanometer (Dinamap Pro Series 100V2, GE Medical Systems). IOP and blood pressure were used to calculate the mean ocular perfusion pressure (OPP) in the sitting position, as follows: OPP = $2\left/ 3\right.$ (mean arterial pressure) – IOP [21], where mean arterial pressure = diastolic BP + $1\left/ 3\right.$ (systolic BP – diastolic BP).

**Fig. 1:**
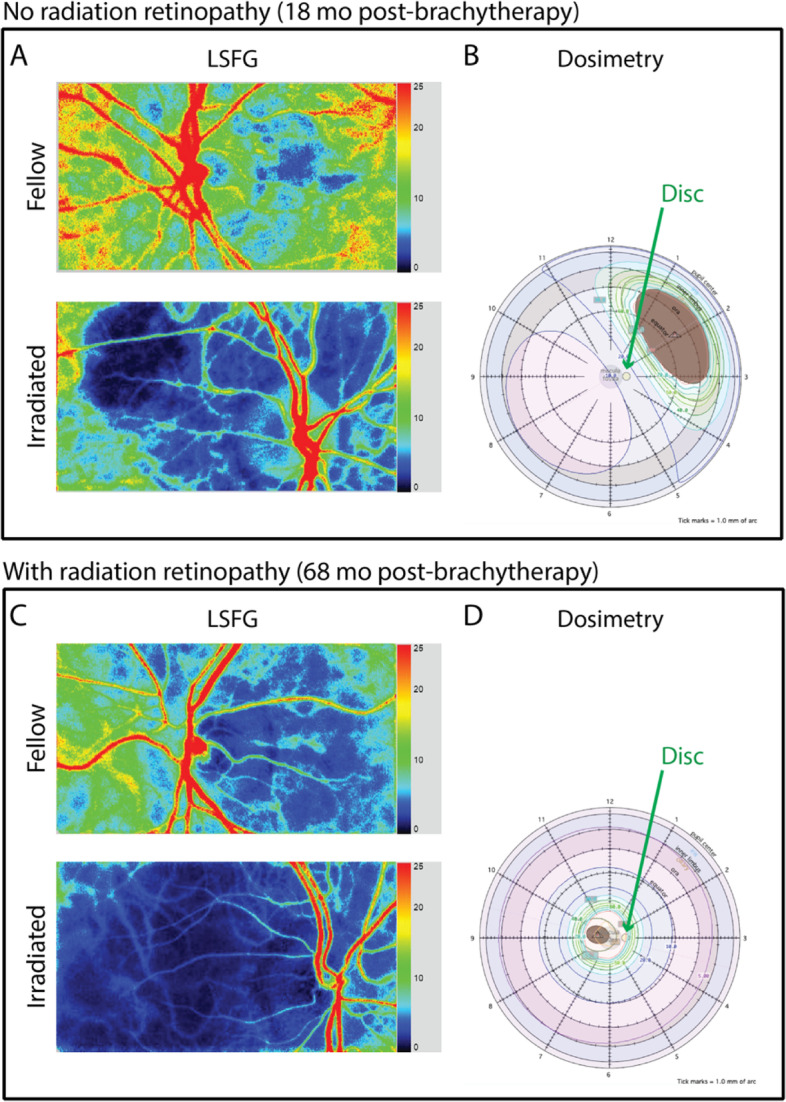
Imaging modalities to assess relative flow changes after.^125^I-plaque brachytherapy. **A**, **C** Laser speckle flowgraphy (LSFG) scans of the optic nerve head and surrounding retina of fellow (top) and irradiated (bottom) eyes. The color scale represents the highest mean blur rate as red and the lowest as blue. **B**, **D** Dosimetry maps constructed to calculate radiation dose to the optic nerve head (indicated by green arrow). Representative images are shown from treated and fellow eyes of a patient without clinical signs of radiation retinopathy (seen 18 months after treatment, top) and with radiation retinopathy (seen 68 months after treatment, bottom) as diagnosed by color fundus photography. Note that in the irradiated eyes the branch retinal arterioles and venules show reduced flow. The large blue (low flow) areas correspond to reduced choroidal blood flow. Dosimetry models constructed using Plaque Simulator (v.6.6, EyePhysics, LLC)

Three successive LSFG scans of the optic nerve head and surrounding retina were acquired in both eyes of each participant using LSFG-NAVI (Softcare Co., Ltd.), a fundus camera coupled with an 830 nm diode laser and a charge coupled-device sensor (750 × 360 px area) as the detector. The principles of LSFG have been previously described in greater detail [22, 23]. Briefly, LSFG utilizes an infrared laser speckle pattern imaged onto the retina. Blood flow at each pixel is derived by calculating the mean blur rate generated by moving red blood cells. Faster movement produces greater blur of the contrast pattern at each pixel and blur rate has been found to be linearly proportional to blood flow and validated by comparison to hydrogen gas clearance [24] and microsphere methods [25]. Analysis of this pattern yields the relative flow velocity, or mean blur rate (MBR), which describes blood flow in arbitrary units (AU). Data used for analysis are acquired over a 4 s period of video recording, at a rate of 30 frames per second, to produce the temporal composite image (Fig. 1A, C) of average blood flow at each pixel. Corresponding dosimetry maps shown (Fig. 1B, D).

**Fig. 2:**
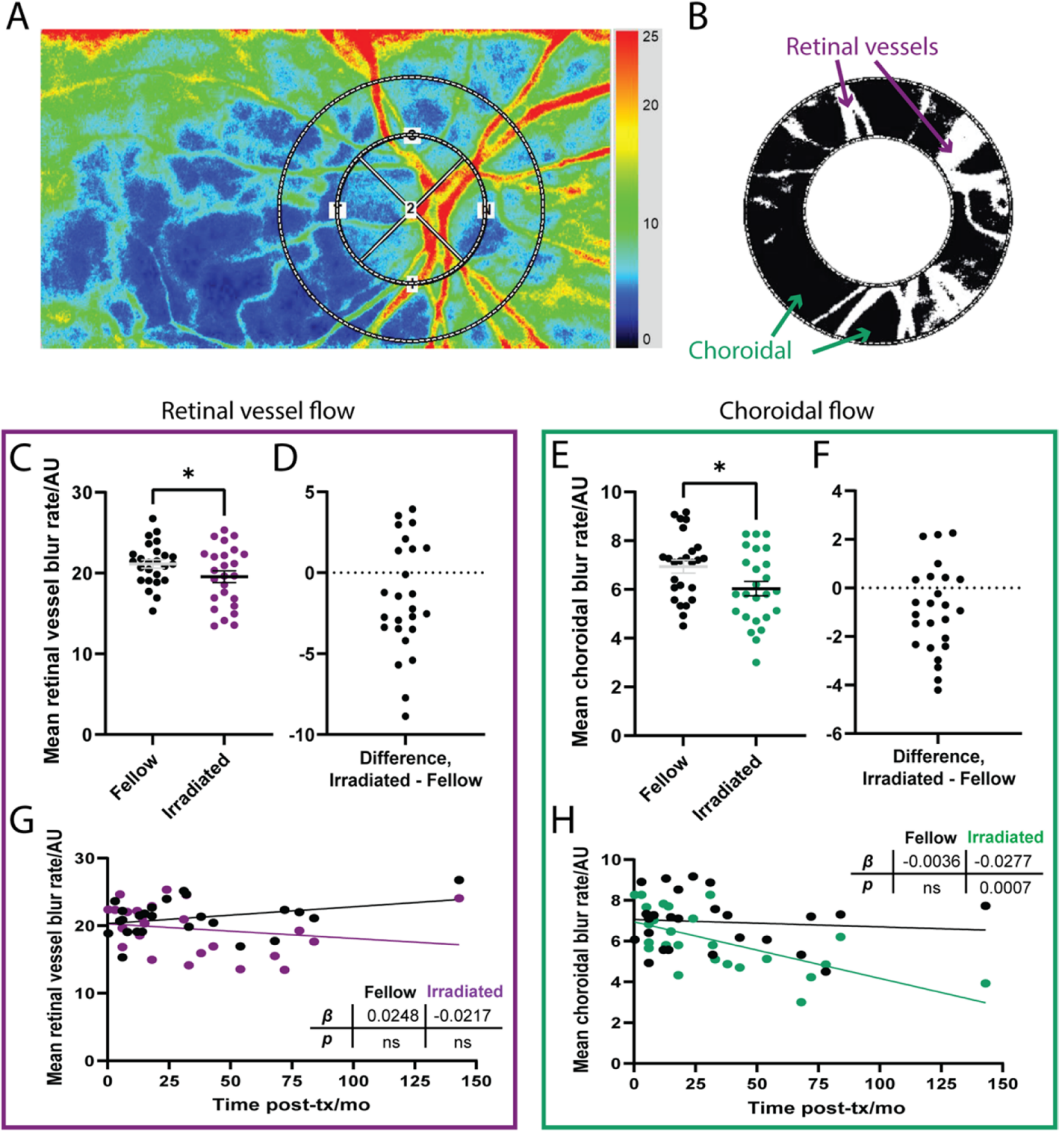
Peripapillary relative blood flow velocity after.^125^I-plaque brachytherapy by standard LSFG analysis. **A** Representative LSFG image with overlaid measurement annuli for analysis. **B** Flow in the annulus is separated into areas primarily representing retinal (white pixels) and choroidal (black pixels) vascular supply following automated histogram thresholding. **C** Pairwise comparison of retinal vessel flow in irradiated (purple) and fellow (black) eyes. **D** Differences in retinal vessel flow between irradiated and fellow eye for each patient. **E** Pairwise comparison of choroidal flow in irradiated (green) and fellow (black) eyes. **F** Differences in choroidal flow between irradiated and fellow eye for each patient. **G** Retinal vessel flow in irradiated (purple) and fellow (black) eyes as a function of time from treatment. **H** Choroidal flow in irradiated (green) and fellow (black) eyes as a function of time from treatment. * p < 0.05 by two-tailed paired t-test (**C**, **F**). Slope (*β*) and its *p* value calculated by simple linear regression (**G**, **H**)

To analyze flow in the peripapillary region, composite images were opened in the LSFG Analyzer software (v. 3.5.0.0; Softcare, Co., Ltd.). Two concentric annuli (“rubber bands”) centered on the optic nerve head were placed on the image to allow measurement of retinal peripapillary flow. The edge of the inner band was set to the margin of the optic disc, and the second band, approximately one optic disc radius, to include the peripapillary retinal vasculature (outer annulus, Fig. 2A). Superficial retinal vessel flow (“MV”) and choroidal tissue flow (“MT”) in the outer ring were identified using the software’s histogram thresholding method, which calculates a threshold above which flow signal is primarily attributed to superficial retinal arterioles and venules (MV; white pixels, Fig. 2B). The mean blur rate of pixels below the threshold (MT; black pixels, Fig. 2B) primarily represents choroidal blood flow. After bands were applied for every eye, scans were processed using the device’s accompanying batch processing software, Cobitos (v.1.0.52.0, Softcare, Co., Ltd.). Ocular perfusion pressure, MV, and MT were tabulated for all scans in a spreadsheet exported to Microsoft Excel.

**Fig. 3:**
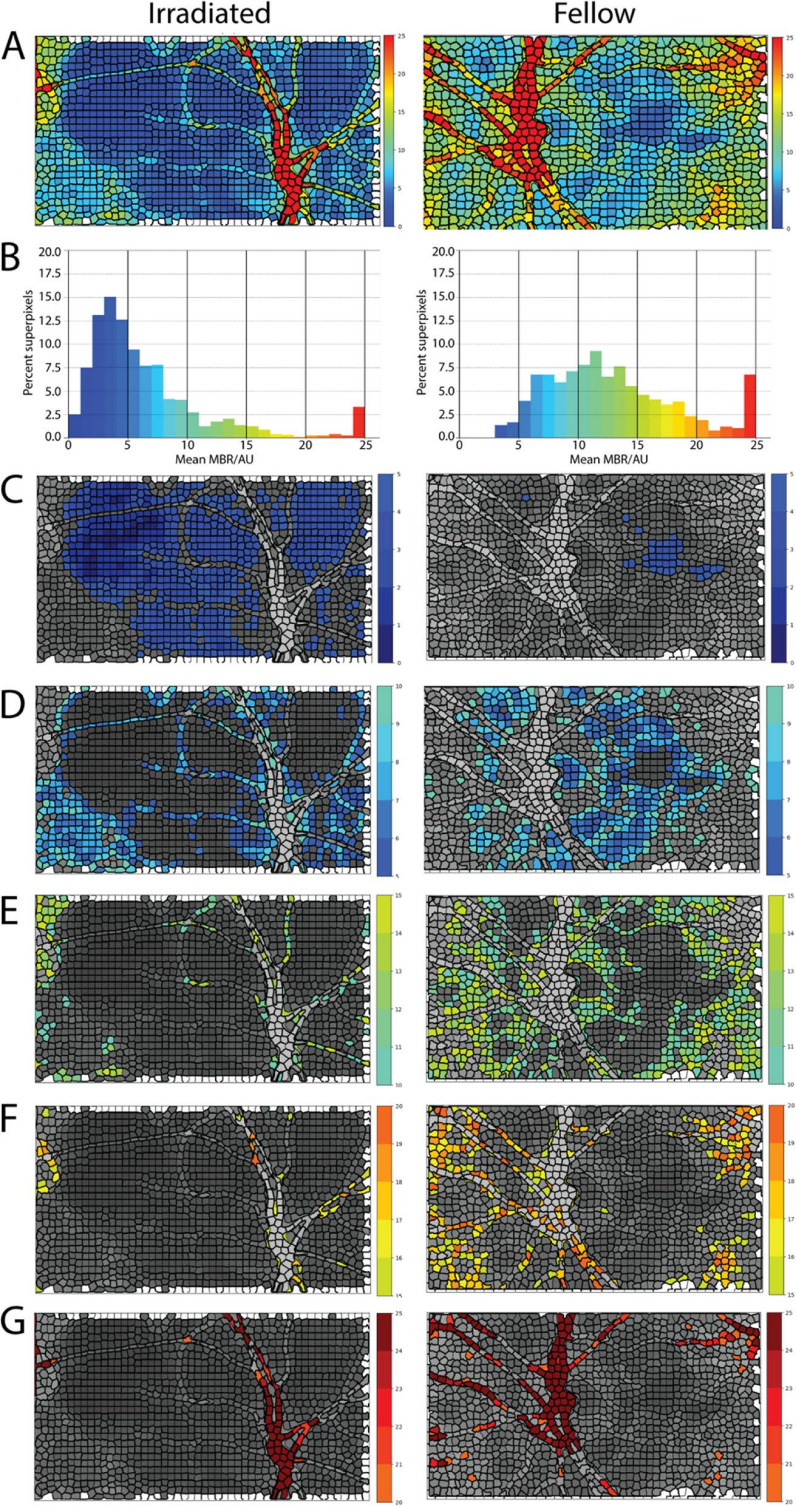
Categories of relative blood flow velocity by superpixel segmentation of the entire scan area. (**A**) Composite maps describing the average mean blur rate (MBR) in superpixels from an irradiated (left) and fellow (right) eye of a patient seen at 54 months post-brachytherapy. The color scale represents the highest mean blur rate as red and the lowest as blue. White superpixels contain undefined values over the 4 s period of data acquisition. (**B**) Histogram of the percent of superpixels with five different ranges of MBR values, following the same color scale as in (**A**). (**C-G**) Composite images highlighting superpixels with a mean MBR < 5 (**C**), a mean MBR of 5 to 10 (**D**), a mean MBR of 10 to 15 (**E**), a mean MBR of 15 to 20 (**F**), and a mean MBR ≥ 20 (**G**). The color scales for (**C-G**) match the colors in (**A**) and (**B**). Note that in this example there is a shift in the percent of superpixels in the irradiated eye away from the highest (red) to the lowest (blue) blood flow range, compared to the fellow non-irradiated eye in both the histogram and the blood flow pixel maps

In a separate analysis, we applied a novel superpixel-based segmentation method to analyze the MBR over the entire scan area. For this purpose, the flow velocity histogram of each composite LSFG scan was segmented using a modified k-means superpixel segmentation method. For each input composite image, a simple-linear-iterative-clustering (SLIC) algorithm [26] was applied to group regular pixels into superpixels. Each image was segmented such that approximately 1500 superpixels were defined per image; the compactness factor, which controls the balance between MBR uniformity and space proximity, was set to 50. Then, the average MBR value in each superpixel was calculated (Fig. 3A). The distribution of flow values across the entire rectangular scan was described by generating histograms of the percentage of superpixels in each MBR categorical range of blood flow (Fig. 3B). Segmented images were generated showing the number of superpixels in the < 5, 5 to 10, 10 to 15, 15 to 20, and ≥ 20 MBR groupings (Fig. 3C-G). The highest MBR range ≥ 20 primarily represents the blood flow in the superficial retinal vasculature, whereas the MBR in the lowest range < 5 primarily corresponds to areas of the lowest choroidal blood flow [27]. Most of the MBR categorical ranges between 5–20 MBR represent a range of choroidal blood flow, as retinal capillary flow supplying the inner retina contributes very little to the total MBR at each pixel located in between the retinal arterioles and venules [28]. Therefore, for the analysis of choroidal and superficial retinal vessel flow, the lowest < 5 and highest ≥ 20 MBR ranges were considered in the superpixel analysis (Fig. 3C and G, respectively).

### Statistical analysis

The average of the measurements obtained from three successive LSFG scans was used for data analysis. Paired, two-tailed t-tests were used to compare data from each patient’s irradiated eye to their fellow eye. Linear regression was used to identify significant variations in each measure with time from treatment. Spearman correlation (two-tailed, 99% confidence interval) was used to identify significant relationships between each outcome measure. All statistical calculations were performed using GraphPad Prism (v.8.2.1 for Windows, GraphPad Software).

## Results

### Participant population

Detailed demographics for the 25 patients enrolled were reported in a previous study [11]. Briefly, the average age of the patients was 61 years, and 68% were male. At the time of imaging, the average time from treatment was 33 months. As per ICD-10 code, 24% had hypertension, 16% had diabetes mellitus, and 8% of patients had both diabetes and hypertension. One patient had been diagnosed with mild diabetic retinopathy by a single microaneurysm in each eye in their color fundus photographs. The average systolic blood pressure was 136 mmHg (Table 1). The average IOP was 14 mmHg in irradiated eyes and 15 mmHg in fellow eyes (*p* ns). Nineteen patients received one or more bevacizumab injections at the time of or after ^125^I-plaque brachytherapy. One patient was treated with bevacizumab 2 weeks before the imaging was performed. In all other patients, the last injection had occurred 2 months or longer before testing (Supplementary Table 1), by which time the effects of bevacizumab on blood flow have been shown to resolve [29].

**Table 1:** Subject demographics

**Features**	**Patients** no. (%), *n* = 25
Systolic blood pressure (mmHg), mean ± SD	136 ± 17
Diastolic blood pressure (mmHg), mean ± SD	80 ± 10
Intraocular pressure (mmHg), mean ± SD	
Irradiated eyes	14.4 ± 3.8
Fellow eyes	15.2 ± 3.2
Ocular perfusion pressure (mmHg), mean ± SD	
Irradiated eyes	51.5 ± 7.9
Fellow eyes	50.7 ± 7.5

A more detailed report of radiation treatment and pre-radiotherapy tumor features for this population was provided in the supplementary materials of our previous study [11]. Briefly, tumors involved either the choroid (64%), the ciliary body (20%), or peripapillary region (within 1 mm of the optic disc; 16%). With respect to the optic disc, tumors were located in the peripheral macula (24%) or in the nasal (20%), superior (16%), superiotemporal (16%), inferior (16%), inferiotemporal (4%), or inferionasal (4%) quadrants. The mean largest basal diameter of the tumor was 10.7 mm; the mean thickness, 2.6 mm. The mean distance from the tumor margin to the foveola was 6.0 mm. The mean distance from the tumor margin to the disc was 5.0 mm, and the average dose to the disc center was 38 Gy (Fig. 1B, D).

### Peripapillary blood flow decreases after brachytherapy

First, we applied a standard method of analyzing retinal LSFG scans, which measures blood flow velocity (MBR) in the peripapillary region (Fig. 2A-B). With this approach, the superficial vascular MBR (MV; retinal arterioles and venules within the measurement annulus) was significantly decreased in irradiated eyes of 17 of 25 patients compared to their fellow eye (*p* = 0.0321, Fig. 2C-D). Similarly, choroidal MBR (MT within the measurement annulus) was significantly decreased in irradiated eyes of 17 of 25 patients in comparison to their fellow eye (*p* = 0.0186, Fig. 2E-F). While superficial retinal flow with this analysis method did decrease with time from brachytherapy, the slope of the trendline (*β*) was not statistically significant (Fig. 2G). Choroidal MBR in irradiated eyes significantly decreased with time from brachytherapy (*p* = 0.0007, Fig. 2H). No significant trends in retinal or choroidal blood flow with time from brachytherapy were observed in the fellow, nonirradiated eyes.

### A superpixel-based analysis detects reduced retinal and choroidal blood flow

**Fig. 4:**
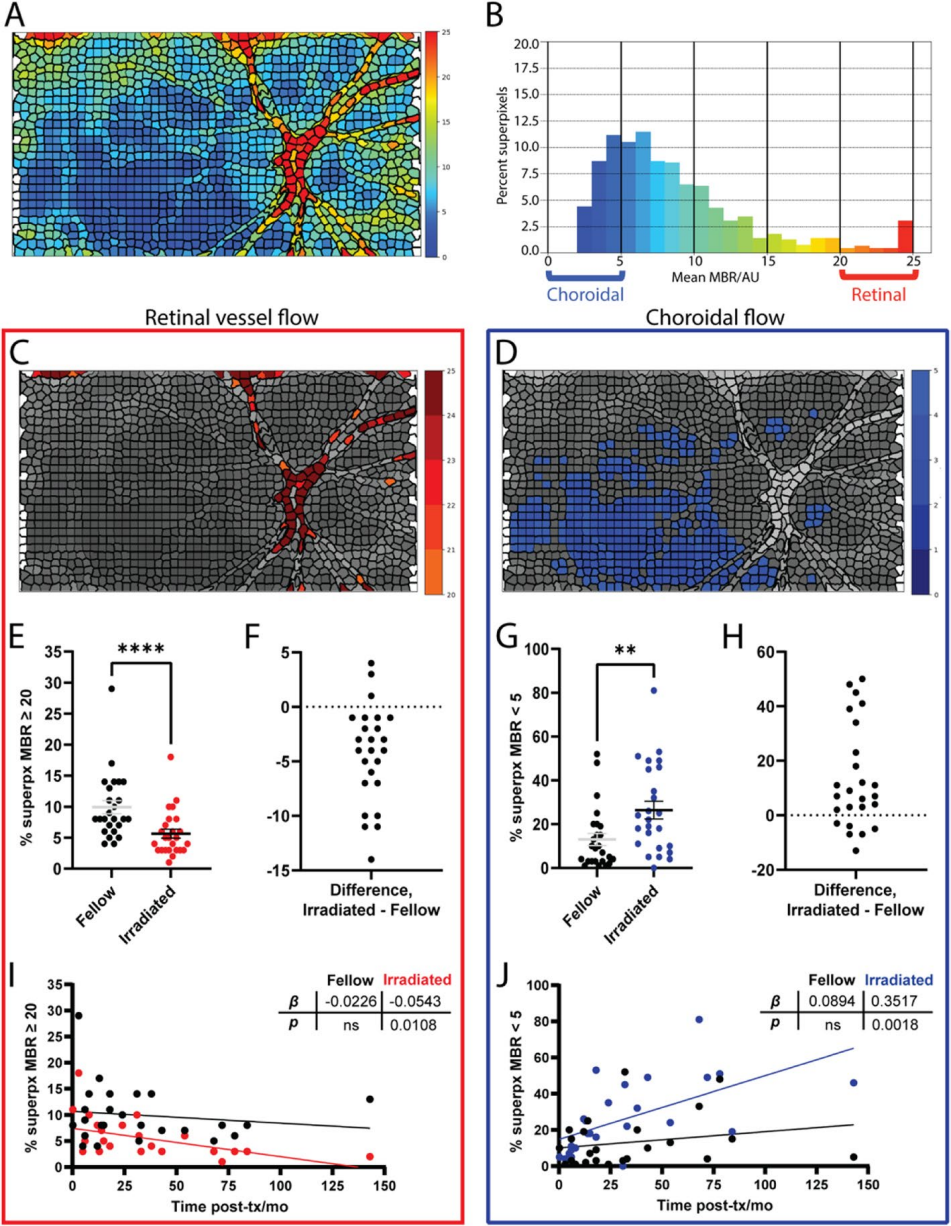
Relative blood flow velocity after.^125^I-plaque brachytherapy by superpixel analysis. (**A**) Representative composite image of MBR by superpixel analysis from the irradiated eye of a patient seen 18 months post-brachytherapy. (**B**) Histogram distribution of percent superpixels for each MBR range in (**A**). Colors match the color scale shown in (**A**). (**C**, **D**) Superpixel segmented composite image of MBR ≥ 20 (**C**) and < 5 (**D**). (**E**, **G**) Pairwise comparison of retinal flow area (MBR ≥ 20, **E**) and choroidal flow area (MBR < 5, **G**) in irradiated and fellow eyes. (**F**, **H**) Differences in retinal (**F**) and choroidal (**H**) flow area between irradiated and fellow eye for each patient. (**I**, **J**) Percent superpixels with MBR ≥ 20 (**I**) and MBR < 5 (**J**) in the irradiated and fellow eyes as a function of time from treatment. ** p < 0.01, **** *p* < 0.0001 by two-tailed paired t-test (**E**, **G**). Slope (*β*) and its *p* value calculated by simple linear regression (**I**, **J**)

To analyze relative blood flow velocity over the entire scan area, we applied a superpixel-based method that analyzes categorical variations in MBR. For each superpixel LSFG scan (Fig. 3A, Fig. 4A), the flow velocity histogram was divided into five ranges of MBR (Fig. 3B, Fig. 4B); color-coded maps were generated for each range (Fig. 3C-G). Flow in the superficial retinal arterioles and venules was associated with the highest ≥ 20 MBR range (Fig. 3G, Fig. 4C); lowest flow was associated with the < 5 MBR range (Fig. 3C, Fig. 4D). By this method, retinal vessel flow area (percent of total pixel area) was significantly decreased in irradiated eyes of 22 of 25 patients when compared to the fellow eye (*p* < 0.0001, Fig. 4E-F). Conversely, the pixel area of lowest flow velocity (percent of total pixel area) significantly increased in irradiated eyes of 19 of 25 patients when compared to the fellow eye (*p* = 0.0017, Fig. 4G-H). Significant differences between irradiated and fellow eyes were also observed for the 10 to 15 and 15 to 20 MBR ranges (*p* = 0.0194 and 0.0281, respectively; data not shown; see Fig. 3E-F for example). When compared to time from treatment, sizeable decreases in blood flow were observed as early as 3 months post-brachytherapy and continued to decrease significantly with time (Fig. 4I-J). The area of lowest flow velocity (Fig. 4J) changed more rapidly and more significantly than the area of highest flow velocity (Fig. 4I; *p* = 0.0018 and 0.0108, respectively). Neither measure in the fellow, nonirradiated eyes varied significantly with time from brachytherapy.

**Fig. 5:**
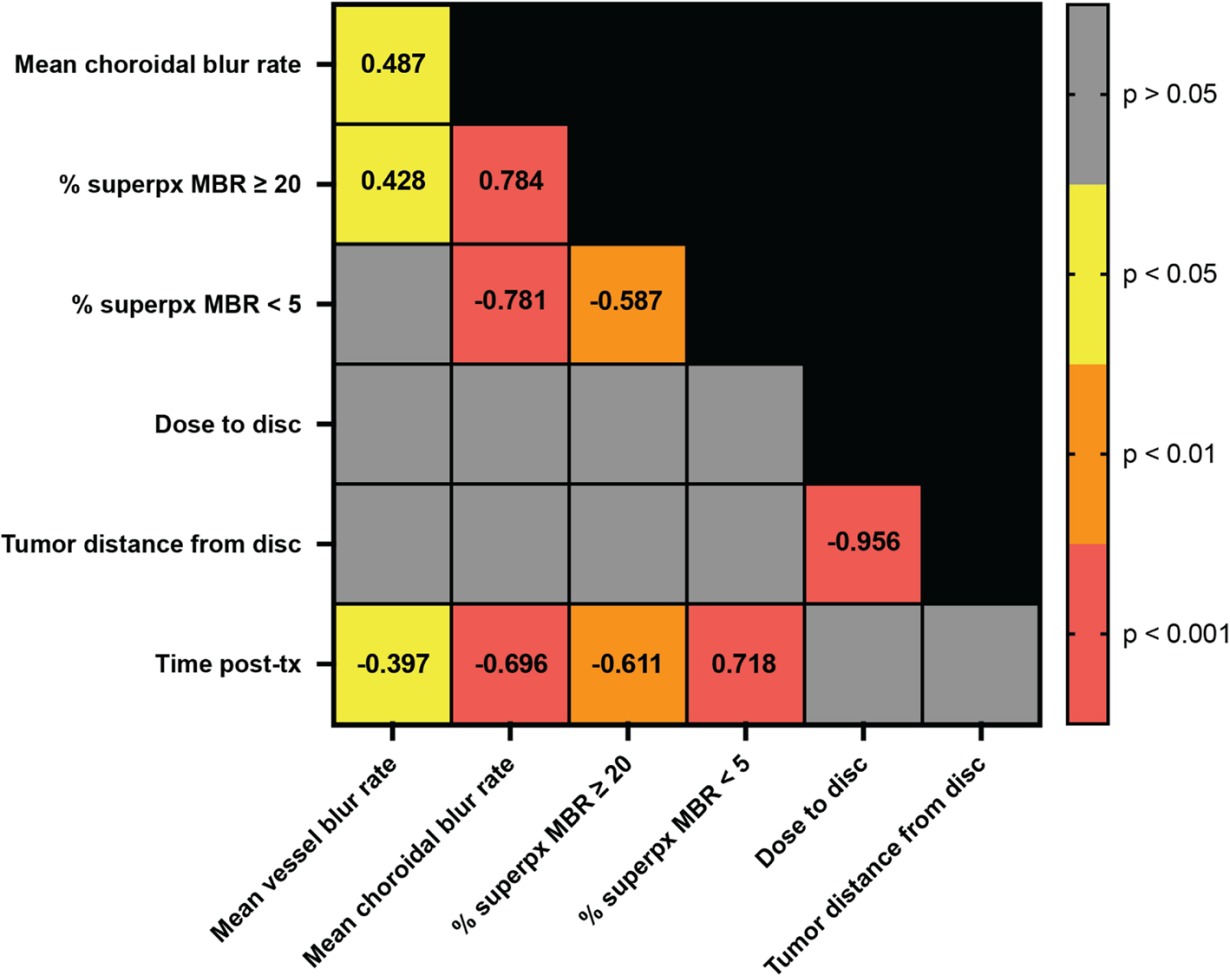
Spearman correlation matrix of readouts. Interrelationships between outcome measures are reported for each statistically significant measure and color coded for degree of significance. Mean choroidal blur rate and mean retinal vessel blur rate describe flow rate within the peripapillary annulus (Fig. 2). The % superpx MBR < 5 and % superpx ≥ 20 describe the percent area of choroidal and retinal flow calculated using a superpixel method (Fig. 4). Distance from the tumor margin to the optic disc, as well as dose to the optic disc, were obtained from clinical treatment planning records

To further substantiate the relationships between the readouts for laser speckle blood flow, radiation dose to the optic disc, and time from treatment, we generated a Spearman correlation matrix (Fig. 5). In agreement with our analysis of the linear regression trendlines, correlations with time from brachytherapy were observed for superficial vascular and choroidal MBR (negative correlation), percent of pixels in the highest ≥ 20 MBR range corresponding to retinal vessel flow (negative correlation), and percent of pixels in the lowest < 5 MBR range corresponding to choroidal flow (positive correlation), with the strongest correlation observed between time from treatment and percent of pixels in the lowest < 5 MBR range corresponding to choroidal flow (*r* = 0.718, *p* < 0.001). No significant correlations were observed between blood flow, radiation dose to the optic disc, nor distance from the tumor margin to the disc.

## Discussion

In this study, we utilized LSFG, a non-invasive method for quantifying relative blood flow velocity, to assess retinal and choroidal blood flow following ^125^I-plaque brachytherapy. Overall, in patients imaged within 24 months after brachytherapy, decreased blood flow compared to their fellow, non-irradiated eye was seen in 13 of 14 patients. Within the first 6 months following brachytherapy, 5 of 6 patients sampled during this time interval had decreased blood flow compared to their fellow, non-irradiated eye. The strongest correlations of altered flow with time from treatment were observed by our superpixel-based measures of blood flow, rather than the standard LSFG analysis of flow around the optic nerve head in the peripapillary region. Importantly, in our analysis, we analyzed the flow in the irradiated compared to fellow eyes for each patient, as an internal control. This comparison should account for any effect of systemic vascular disorders like diabetes meillitus or hypertension, which is expected to affect both eyes equally. Overall, these findings suggest decreased relative blood flow velocity measured by LSFG may be useful as a clinical marker for early microvascular response to radiation therapy.

Established LSFG analyses focusing on disorders of the optic nerve head have measured flow velocity in the larger retinal arterioles and venules (MV) located on the optic nerve head. In the case of radiation retinopathy, focal pathology may develop first in distal retinal vessels that may only represent a small part of the area supplied by larger proximal arterioles. To improve detection of regional decreases in blood flow, we applied a superpixel analysis method, which allowed us to expand our blood flow analysis to the entire scan area and discern flow in all arterioles and venules (% superpx MBR ≥ 20) and choroidal vascular beds with the lowest flow (% superpx MBR < 5). Compared to the traditional published LSFG analysis of retinal arteriole and venule velocity, the superpixel-based analysis revealed more significant decreases in blood flow with time from brachytherapy. The superpixel LSFG analysis method may also be advantageous for assessing regional changes in retinal and choroidal blood flow in other retinal microvascular disorders such as diabetic retinopathy, where decreases in blood flow may precede capillary dropout across the retina [30].

The main findings of this study are consistent with Doppler and photomicrography imaging studies in other vascular beds such as skin and parotid glands in irradiated small animals, where progressive reductions in blood flow were observed within hours of radiation exposure followed later by capillary closure [14–16]. Studies of irradiated monkey eyes and rabbit ear chambers have also shown closure of the smallest blood vessels, often preceded by deceleration of blood flow [15, 31]. Here, in accordance with these findings, we observed the most significant decreases in blood flow in regions reflecting blood flow in the choroid [27, 28]. The relationship with time from brachytherapy was strongest and most significant for the superpixel analysis corresponding to choroidal flow (*β* = 0.3517, *p* = 0.0018) and the superpixel analysis corresponding to retinal vessel flow (*β* = -0.0543, *p* = 0.0108). A longitudinal study deploying both OCT-A and LSFG will help to confirm decreases in blood flow prior to capillary closure and vessel loss.

One limitation of our study is that the anti-VEGF agents frequently given after ^125^I-plaque brachytherapy [32–34] decrease retinal blood flow. In fact, several recent studies have deployed LSFG and reported reduced retinal perfusion, but which resolved by 2 months after injection [29, 35, 36]. Of note, in our cohort, only 1 patient was treated with bevacizumab less than 2 months before imaging. Thus, we are confident that the findings herein are directly related to ^125^I-plaque brachytherapy rather than side effects of anti-VEGF therapy. Another limitation is that the LSFG signal attributed to superficial retinal arterioles and venules also includes some signal from the underlying choroidal flow. However, when in an additional analysis, the underlying choroidal flow was removed from the peripapillary region, 4 of the 6 patients imaged within the first 6 months after radiotherapy had decreased blood flow compared to the contralateral eye (data not shown). This would support our interpretation of a decrease in flow in the superficial retinal arterioles and venules.

## Conclusions

In summary, we developed a novel approach to analyze large scan areas in LSFG by a superpixel-based analysis. The application of this method to a cross-sectional cohort of patients with uveal melanoma after ^125^I-plaque brachytherapy revealed reduced retinal and choroidal blood flow as an early indicator of microangiographic injury after radiation therapy. This offers the opportunity for early detection and intervention to mitigate the long-term vascular sequelae and potentially to monitor its response to treatment. Further application of this method to a longitudinal cohort in which repeat LSFG measurements in tandem with OCT-A imaging are performed is anticipated to provide a more comprehensive insight into the relationship between temporal decreases in flow to complete loss of capillary flow.

## Abbreviations

BP: Blood pressure
IOP: Intraocular pressure
LSFG: Laser speckle flowgraphy
MBR: Mean blur rate
MT: Choroidal tissue flow
MV: Superficial retinal vessel flow
NS: Nonsignificant
OCT-A: Optical coherence tomography-angiography
OPP: Ocular perfusion pressure
SD: Standard deviation
SLIC: Simple-linear-iterative-clustering
VEGF: Vascular endothelial growth factor
